# Reimagining Partnerships Between Black Communities and Academic Health Research Institutions: Towards Equitable Power in Engagement

**DOI:** 10.3390/ijerph22060921

**Published:** 2025-06-10

**Authors:** Khadijah Ameen, Collins O. Airhihenbuwa, Kimberley Freire, Monica Ponder, Alicia Hosein

**Affiliations:** 1 Department of Policy and Behavioral Sciences, School of Public Health, Georgia State University, 140 Decatur St, Atlanta, GA 30303, USA; cairhihenbuwa@gsu.edu (C.O.A.); kfreire@gsu.edu (K.F.); ahosein1@student.gsu.edu (A.H.); 2 Department of Communication, Culture and Media Studies, Cathy Hughes School of Communications, Howard University, 300 Bryant St NW, Washington, DC 20059, USA; monica.ponder@howard.edu

**Keywords:** community-academic partnerships, community-engaged research, health equity, community power, black populations

## Abstract

Community-Engaged Research (CER) arose as a means of increasing the democratic participation of communities that study outcomes directly impact. CER has been identified as a recommended approach for conducting biomedical and behavioral health research with Black communities, a population that has been excluded from and exploited by academic health research for centuries. However, solely increasing community participation without identifying and redressing racialized power imbalances within community–academic partnerships involving Black populations can stall progress towards racial health equity. The purpose of this study was to explore how power can be redistributed equitably in community–academic health research partnerships involving Black populations. Utilizing the qualitative methodological approach of critical narrative inquiry, counter-stories from 12 Black individuals who have served as community partners on U.S.-based academic health research teams were collected via in-depth semi-structured narrative interviews. A reflexive thematic analysis approach was utilized to identify and analyze strategies expressed by study participants for increasing community agency, efficacy, and solidarity in health research. By centering the voices of Black community members who have directly engaged with academic health research institutions, this study sought to amplify the desires and aspirations of Black communities regarding shifting power in health research processes and outcomes.

## 1. Introduction

Community-Engaged Research (CER) arose as a means of increasing the democratic participation of communities that study outcomes directly impact [1]. CER is a set of approaches to research that diverges from dominant top-down research approaches in which academic research institutions own, manage, and dictate entire research processes and outcomes. The level of community engagement across CER approaches ranges across a continuum. On one end of the CER continuum are community-informed research approaches that minimally engage community members through one-way communication channels [2,3]. On the other end of the CER continuum are community-based participatory research approaches that emphasize bi-directional communication and the leadership of community members across all aspects of the research process and outcomes [2,3]. A common form of CER lies in the middle, whereby community members serve in a consultative, advisory capacity to the research projects owned and managed by academic research institutions [2,3].

CER has been identified as a recommended approach for conducting health research with Black communities, a population that has been excluded from and exploited by academic health research for centuries due to medical and scientific racism [4,5,6,7]. However, efforts to increase community participation without attempting to redress racialized power imbalances within community–academic partnerships (CAPs) involving Black populations has stalled progress towards racial health equity [8]. As social work scholar Sherry Arnstein describes, “participation without redistribution of power is an empty and frustrating process for the powerless. It allows the power-holders to claim that all sides were considered, but makes it possible for only some of those sides to benefit. It maintains the status quo” [9] (p. 216). Participation without intentionally challenging dominant racialized power arrangements in which academic research institutions maintain power leads to community partnerships that are merely symbolic, ritualistic, performative, and tokenistic rather than justice-oriented [9,10,11]. Black community members involved in these forms of CER projects may be granted the opportunity to hear (e.g., through a community-informed capacity) and be heard (e.g., through community advisory capacity). However, power over research agenda-setting, decision-making across the research lifecycle, and research ideologies often remain concentrated within and between academic research institutions [10,12]. This top-down, university-managed approach relegates Black community partners to be invited guests in the research spaces of academic research institutions. As guests, they are limited in their ability to fully co-create knowledge, equitably access material resources and capital, and culturally define themselves within that space [13,14].

The second critique is of the shortcomings of many CER projects that do seek to move beyond participation to empowerment. According to Israel and colleagues, empowerment generally refers to “the ability of people to gain understanding and control over personal, social, economic, and political forces to take action to improve their life situations. In contrast to reactive approaches that derive from a treatment or illness model, the concept of empowerment is positive and proactive” [15] (p. 152). Empowerment can occur across different levels of analysis and contexts, from the individual level to the organizational and community level [15]. While CER practices at academic health institutions have largely moved away from power arrangements that exercise “power over” Black research participants (e.g., through oppression, control, exploitation, domination, coercion, and violence), academic health research institutions commonly wield “power for” research participants as an attempt at empowerment [11,16,17,18,19]. “Power for” arrangements involve “those with hierarchical power choosing to exercise it beneficently on behalf of others” [11] (p. 4). This arrangement is commonly expressed in research processes when academics give themselves the right to speak for Black communities.

Academic health researchers engaged in CER with Black communities may fall into the trap of dictating the terms of empowerment for such communities, engaging in top-down approaches, one-way communication, and vertical relationships with community partners [20]. These roles are often (1) reactionary rather than proactive in nature; (2) externally motivated by financial, professional, and social capital; and (3) inherently racialized, perpetuating white saviorism, paternalism, and racial domination [20,21,22,23]. This “power for” arrangement may also appear as health equity tourism, a phenomenon conceptualized by Lett et al. as “the practice of investigators—without prior experience or commitment to health equity research—parachuting into the field in response to timely and often temporary increases in public interest and resources” [22] (p. 17). This arrangement is also informed by a concept referred to as interest convergence, a Critical Race Theory principle advanced by legal scholar Derrick Bell that refers to the phenomenon when the material interests of a group in power align with the social justice priorities of a minoritized group [21,24]. Inattentiveness to redressing power dynamics and the false appearance of empowerment can lead to the further disempowering of Black communities already harmed by academic health research institutions [20].

The purpose of this study was to explore how power can be redistributed equitably in community–academic health research partnerships involving Black populations. Utilizing the qualitative methodological approach of critical narrative inquiry, counter-stories from 12 Black individuals who have served as community partners on U.S.-based academic health research teams were collected to uplift community-based desires and needs regarding equitable health research engagement.

Critical narrative inquiry is an approach that generates insights by examining the interplay of everyday personal narratives, interpersonal relationships and interactions, institutional discourses, and societal power arrangements through stories [25,26]. Counter-storytelling is a critical race methodology and a specific form of critical narrative inquiry that intentionally centers the voices of marginalized racial and ethnic people [27]. By centering Black community members who have directly engaged with academic health research institutions, this study seeks to locate and amplify the voices of Black communities when engaging in community–academic health research partnerships.

**Figure 1:**
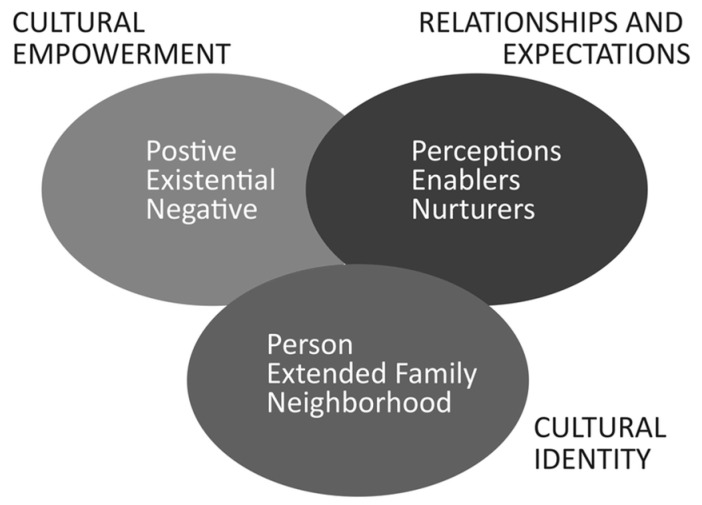
PEN-3 Cultural Model.

This study is theoretically underpinned by the PEN-3 Cultural Model (Figure 1). Culture can be defined as “the shared values, norms, and codes that collectively shape a group’s beliefs, attitudes, and behavior through their interaction in and with their environments” [28] (p. 2). Culture is central to understanding health beliefs and outcomes and is foundational to understanding how groups of people contribute to, resist, and transform their relationships to power in society [29]. The PEN-3 Cultural Model (PEN-3) offers a framework for understanding the various ways in which cultural context shapes health beliefs, behaviors, and outcomes [30,31]. The model challenges Eurocentric, Western paradigms of understanding the connection between culture and health for minoritized groups [31]. The PEN-3 Cultural Model consists of three primary domains: (1) Cultural Empowerment, (2) Relationships and Expectations, and (3) Cultural Identity [30,31]. The Cultural Empowerment domain is particularly relevant to the research project. A central theme of PEN-3 is that culture should be understood beyond a deficits-based framing (cultural barriers) and should encompass both positive (cultural strengths) and existential (unique opportunities) dimensions. This study uses a strengths-based lens to center the collective cultural power, resources, and capabilities that Black communities already possess to create radical healing spaces for framing health problems and generating solutions through research [32]. This study also explores unique opportunities for building community efficacy to more equitably engage in health research processes. This study is informed by the Relationships and Expectations domain of PEN-3, particularly as it pertains to the relational power dynamics and solidarity between academic health researchers and Black communities. Finally, this study is informed by the Cultural Identity domain of PEN-3, with specific attention to how self and community determination, definition, and agency are critical to advancing Black communities’ equitable engagement in biomedical and behavioral health research.

## 2. Materials and Methods

### 2.1. Sampling Strategy and Participant Criterion

A purposive sampling strategy combining criterion-based and snowball methods was used to identify research participants. Digital recruitment materials were disseminated via the principal investigator’s (KA) community-based networks to invite potential research participants to the study, including through email and social media channels. The digital recruitment flyer included a QR code and link to an eligibility assessment form on Qualtrics to assess eligibility for individuals interested in participating in the study. Additional research participants were recruited through snowball sampling. The recruitment materials included language about sharing the study information across the recipients’ existing networks. KA invited existing research participants to share study recruitment information with their network.

Prospective research participants were asked to participate in one initial individual 90 min semi-structured narrative interview and one 60 min follow-up member-check focus group. A total of 12 research participants participated in individual interviews, and 9 of the 12 initial research participants participated in follow-up focus groups. All 12 research participants were 18 years old or older, identified as Black or of African descent (e.g., African American, Afro-Caribbean, Afro-Latinx), and resided in the United States (see Table 1). Two participants self-identified as multiracial or multiethnic, identifying as both non-Hispanic Black and an additional race or ethnicity. All research participants served as community partners (e.g., community advisory board members, steering committee members) on a health-related research study at an academic institution.

### 2.2. Data Collection

Data collection activities occurred over the course of a six-month period (January 2024–June 2024). All interviews and focus groups were conducted virtually over the Zoom video-conferencing platform and facilitated by KA. The interview guide was informed by the literature review and the PEN-3 Cultural Model, including questions related to (1) how participants defined themselves, the communities they belonged to, and what Black community means to them; (2) participants’ motivations for getting involved in health research, why improving the health of Black communities was important to them, and their perceptions of Black people’s relationship to health research; and (3) participants’ visions for how their communities could have more power in shaping health research processes and outcomes (see Table 2).

Member-checking (participant validation) was completed with 9 of the 12 participants for credibility and trustworthiness with research participants via four focus group sessions [33]. During the focus groups, KA shared initial findings and participants provided feedback on any modifications to data interpretation. Each research participant received a USD 45 gift card for interview participation and an additional USD 25 gift card for focus group participation. The study was reviewed and approved by the Georgia State University Institutional Review Board prior to commencing data collection activities.

### 2.3. Data Analysis

A reflexive thematic analysis (RTA) approach was utilized to analyze data. RTA is a qualitative data analysis approach that emphasizes the researcher’s active role in co-constructing the meaning of study data along with research participants [34]. RTA involves a six-phase analytical process: familiarization with data, generating initial codes, generating themes, reviewing potential themes, defining and naming themes, and producing a report [35]. Following data cleaning of transcripts to remove personal identifiers in phase I, KA worked with a second coder (AH) to generate initial codes and emerging themes in phases II and III and ensure the process of condensing data into codes was iterative, malleable, and reflexive while also aligned with themes that reflected the voices of research participants. Phase IV involved member-check focus groups with research participants to verify and modify themes. Phase V involved creating conceptual definitions for each theme, and phase VI involved manuscript development led by KA.

A blended inductive and deductive approach was used to analyze data. The data condensation process was both data-driven, guided by the research participants’ counter-stories, and theory-driven (deductive), guided by PEN-3. In analyzing interview data, KA and AH examined individual and community identity formation, relational power dynamics, and proposed community empowerment strategies within and between research participants’ counter-narratives.

### 2.4. Quality Assurance and Researcher Reflexivity

To ensure credibility in the qualitative research process and product, the research team took several internal validity measures. To ensure the data collection instruments (interview guide, Table S1, and focus group guide, Table S2) aligned with the study purpose and research questions, a draft version of the interview guide was shared with critical public health subject matter experts CA, MP, and KF. In addition, three Black community-based practitioners who had worked with academic health research institutions also reviewed the interview guide to provide input on accessibility and cultural responsiveness [36]. To engage in sense-checking, a secondary coder was recruited (AH) to collaboratively seek richer data interpretations. Member-check focus groups were conducted with research participants to co-construct meaning and ensure that the researcher’s interpretations were reflective of participants’ realities. Finally, KA kept an analytic memo to track her decision-making trail throughout the data collection and analysis process.

Consistent with the qualitative approach utilized for the study, KA engaged in researcher reflexivity throughout the research process. At the start of each interview, she spent a few minutes making a positionality disclosure to research participants. She disclosed that while she identifies as Black and of African descent, that Blackness is expansive and thus every Black person’s lived experience is unique. She shared that she holds a hybrid researcher–community position as both an academic and as a director of a community-based organization, and how this hybrid position directly influenced her motivation to pursue this research topic. She also revealed her intersecting social identities, paradigmatic orientation, and disciplinary training, and how those positions and views provided her with varied points of power, privilege, and bias. Outside of the positionality disclosures, KA kept a reflexive journal throughout the duration of the study. In this reflexive journal, she recorded observational field notes from conversations with other Black scholar-activists and documented personal reflections of her experience as a hybrid researcher with an insider–outsider lens.

## 3. Results

Themes are organized in the results section by the three PEN-3 Cultural Model domains: Cultural Empowerment (Domain 1), Relationships and Expectations (Domain 2), and Cultural Identity (Domain 3).

### 3.1. Debunking Cultural Deficits Framing (Domain 1: Cultural Empowerment)

*“[A] common belief from White researchers is that Black people just aren’t interested in or capable of being a part of health research.”*

Three common misconceptions about Black people’s relationship to health research were expressed across participants. The first was that Black people are not interested in or capable of being a part of health research. Participants countered this misconception by sharing that Black people do want to participate in health research and hold leadership positions in health research, but are systemically excluded due to racism. The second misconception mentioned by participants was that Black people are perceived in White dominant society (and in White dominated academic health research) as inherently aggressive, violent, criminal, and inferior. However, participants countered these negative views of Blackness by describing Black people as strong, prideful, and loving.

*“I feel like why we ended up getting excluded from so much research is that they just don’t want to do the work of acknowledging all of the barriers.”*

The third misconception shared was the perception that Black research participants are challenging to engage, difficult to recruit, non-compliant, and hard-to-reach. Participants refuted this argument by offering a counter argument that academic health research opportunities are hard-to-reach and largely inaccessible to Black communities, emphasizing the need for more culturally responsive and equitable strategies for engaging Black populations in health research.

### 3.2. Reimagined Health Research Topics (Domain 1: Cultural Empowerment)

*“It’s about being able to give voice to your desires and pleasures or needs or wants* *.”*

Participants offered a range of topics for their dream health research, from various health conditions (e.g., cancer, mental and behavioral health, reproductive and sexual health) to specific sub-populations within Black populations (e.g., older Black adults, Black women, Black youth). A common thread across responses were health topics informed by personal and community lived experiences with the selected topic. Another commonality was a desire to study Black life rather than Black death. Through a strengths-based lens, several participants expressed an interest in researching Black vitality, joy, pleasure, and intra-communal relationships over Black ailment.

### 3.3. Reimagined Health Research Design and Processes (Domain 1: Cultural Empowerment)

*“That’s all it boils down to, like how are we educating communities directly, I think it does have to come from us, the Black researchers, the Black professionals, because there’s so much mistrust within our community.”*

Participants described a variety of transdisciplinary and community-based data sources and mixed methodologies for their dream health research. Across participants, there was a desire for ensuring that research products were accessible and community-facing, leveraging stories, the arts, and digital media. There was also an expressed interest in more collaborative research design, implementation, and evaluation processes. This collaboration included better ways of circulating and exchanging knowledge beyond academic conferences and peer-reviewed manuscripts, as well as strategies for transferring, adapting, and scaling best practices for racial health equity implementation science. Finally, several participants articulated a desire for health research designed for Black people and by Black researchers.

### 3.4. Reimagined Health Research Outcomes (Domain 1: Cultural Empowerment)

*“I feel like researchers shouldn’t just conduct research, they should also be prepared to take measures that would improve the reason they were there.”*

*“Not just getting information but providing solutions.”*

Participants overwhelmingly wanted to engage in research that moved beyond acknowledging the existence of racial health disparities. Participants were more interested in engaging in research that focused on designing and piloting racial health equity interventions. Regarding interventions, most participants articulated a desire to translate their dream health research into social action to inform community health improvements, provide direct services, increase healthcare access, and change policies.

### 3.5. Reimagined Resources Needed (Domain 1: Cultural Empowerment)

*“Financial. I need money to survive to eat, like, I need to pay people. I believe in paying people equitably for their experiences, not just their education as well.”*

When asked about the resources it would take to conduct their dream health research, participants cited financial resources and human resources. Regarding financial resources, participants were far less interested in increasing their personal financial capital and more interested in equitable distribution of money and other materials resources to Black research participants, Black research institutions (like Historically Black Colleges and Universities), and Black communities overall. Concerning human resources, participants were interested in research team members that were more than subject matter experts or methodological specialists. Participants valued individuals who were passionate and dedicated to advancing racial health equity.

### 3.6. Health Research Motivation (Domain 2: Relationships and Expectations)

*“I come from people who worked for the improvement and the betterment of Black folks, whatever that look like… so I follow in those footsteps.”*

Participants overwhelmingly expressed a sense of personal responsibility to get involved in health research. The rationale for personal responsibility varied, from feeling like a leader in their community, to feeling tired of being under-represented, to wanting to leverage their power and privilege for community benefit. Shared responsibility also appeared as a motivator for health research involvement. This emerged from wanting to contribute to the collective power of past and present Black health researchers and feeling beholden to cultivating the next generation of Black health researchers.

*“I had this motivation to be involved when it comes to research…because of my mom. Because of that [health] situation, I wouldn’t want any other person to ended up in that dire situation.”*

Another overarching motivator was lived experiences with health challenges at the personal, familial, and communal levels. Navigating healthcare barriers personally, having family members deal with complications from infectious and chronic diseases, and witnessing racial health inequities in their own communities were all driving forces for involvement in health research.

*“The first step in getting to a self-sustaining Black society is being healthy. Because if we’re struggling with diabetes and cardiovascular disease and all these things, you know, preventable or not preventable, unmanaged mental illness, I don’t think we can be as great as we could be.”*

Finally, increasing self-efficacy and community efficacy were influential in participants’ decisions to get involved in health research. For some participants, there was a desire to cultivate their personal knowledge and skills to conduct research. For others, there was a desire to increase the capacity of Black communities to conduct and lead health research.

### 3.7. Reimagined Community Role (Domain 2: Relationships and Expectations)

*“Hopefully, I pray I become leader of a research team one day.”*

Most participants expressed a desire to hold a leadership role, such as principal investigator or co-investigator, in their dream health research. This contrasts with dominant community–academic health research partnerships in which academic researchers hold, manage, and determine leadership roles.

*“I would like to have my hands in it. So I would get to be engaged with the living data of how people would utilize… like what would it look like for them to practice this theory in real time.”*

There was also an expressed desire across participants to be in a role that was hands-on across the research lifecycle. This included coordinating and connecting research participants to health resources and services. Finally, several participants expressed wanting to hold different roles throughout their research career, including principal investigator, research participant, research assistant, advisor, and funder.

### 3.8. Reimagined Academic Role (Domain 2: Relationships and Expectations)

*“They should give us money because they have so much. They should also I would say…[provide] guidance or like technical assistance if needed. Not an overseer, not a leader. I really think like, if I need you, if I needed to call to get an SPSS package, I could, and they could send me a link and I can download it for free.”*

Conversely, when asked what role they would want academics to play, most participants expressed a supporting role. Support ranged from providing technical expertise on particular research methods and research product development strategies, to offering subject matter expertise on a specific disease state based on their disciplinary training. Support also looked like serving as a resource, leveraging their social positions and power as academic gatekeepers to redistribute institutional resources like funding and technology.

*“I think it would be important to have an investment from academic researchers…who are invested in the outcome. So being able to have researchers who see the importance of providing ways of thinking and practice towards liberation and freedom. If you are aligned with that, then yes, it would make sense that you would be part of this research. You want to do more of the community work, it would make sense that you would participate.”*

Participants expressed a desire for academic researchers to be in solidarity with their community-based endeavors. This included physically and metaphorically stepping outside of the “ivory tower” to support community coalitions and provide education on health research opportunities and benefits. This also included a requirement for academic partners to have unwavering commitments to racial justice and health equity.

### 3.9. Black Identity (Domain 3: Cultural Identity)

*“Being Black is not a monolith. We are all so vastly different.”*

Among participants, their Black identity was a central social identity for them. However, several other intersecting social identities were disclosed as critical to their identity formation. These included religious affiliation, regional identity, disability status, gender identity, sexuality, immigrant identity, class status, parental identity, and vocational and academic identity. For example, Black womanhood was a central interlocking social identity for several participants.

*“[I’m] kind of grappling in a space of being in between not having the privileges of other people, but definitely having more privileges than the people in the same—I guess if you want to look at the basement metaphor of intersectionality—in the same basement as me.”*

As it relates to vocational and academic identities, several participants expressed holding an insider–outsider status through either current or past affiliations with academic health research institutions. This hybrid status granted differential modes of power and privilege for some participants. Participants that were graduate-level-educated, had formal disciplinary training in the field of study, and/or who were actively affiliated with academic institutions expressed having more decision-making power in their community–academic research partnerships than participants without the same credentialing.

### 3.10. Black Community Belonging and Meaning-Making (Domain 3: Cultural Identity)

*“When I hear Black community, the first thing that comes to my mind is its strengths. Our Black community is mostly based on intellect, strength. And we have this spirit of togetherness, you know… we’re proud about what we know is right.*

Participants had varied positive, negative, and unique perceptions of what Black community meant to them. Some participants attributed positive characteristics when they thought of Black community, such as Black unity, Black pride, Black love, and Black strength.

*“It really is a lot of like unity, but there’s also so much division as well… [there are] so many things that are being used as dividers in our community, whether it’s colorism, whether it’s sexual orientation, whether it’s cis, trans, there’s a lot out there.”*

Others expressed intra-communal division and stigma within Black communities that alienate Black community members with multi-marginalized identities. Several participants noted that Black people are a socially disadvantaged group that is systemically denied the opportunity to reach full potential due to racism.

*“I’m African descent. I was born and raised here. My parents are from Cameroon. So for me just having that identity…am I American and am I African enough? Am I Black enough?”*

While many participants noted shared Black experiences, others pointed out the unique nuances within and between Black people across the diaspora. Black communities (plural) were described as differing based on social, cultural, political, temporal, and geographic contexts.

## 4. Discussion

Study results exemplify the desire of Black community partners to have more equitable power in health research processes and outcomes. This is aligned with existing literature on community power. While community participation in health research is a critical step to redressing the legacy of racism experienced by Black research “subjects”, racial health justice cannot be actualized without redistributing research-related resources and decision-making authority to advance community power [37]. Shifting and building power at the community level requires movement away from asymmetrical arrangements in which dominant groups can wield “power for” and “power over” the destinies of minoritized groups [11,14,16,18,38]. Power building at the community level operates beyond individual-empowerment models common in the health sciences that aim to build the self-efficacy and resilience of minoritized individuals to adapt to structural oppression. Building community power involves “the ability of communities most impacted by inequity to act together to voice their needs and hopes for the future and to collectively drive structural change, hold decisionmakers accountable, and advance health equity” [39].

Community empowerment (a critical focus area of the PEN-3 Cultural Model) is an emancipatory process in which minoritized communities collectively act to transform health-harming community conditions and advance common good [28,37,40,41,42]. This form of emancipatory power enhances the collective capabilities of communities to harness community agency (“power to”), community efficacy (“power within”), and community solidarity (“power with”) [11,14,17,40,43]. Community agency is the right to exercise agency to improve community conditions through collective authority and social action [40]. Community efficacy is the internal community confidence, self-determination, and critical consciousness necessary to collectively make community change [40]. Community solidarity involves coalition-building and the alignment on shared values, interests, and alliances between partners needed for social mobilization and the advancement of social justice imperatives [40]. In community-engaged health research, this would manifest as (1) Black communities having increased agenda-setting power, decision-making power, and ideological power across research processes and outcomes; (2) Black communities being equipped with the necessary resources, knowledge, skills, and infrastructure to conduct health research; and (3) academic health researchers and research institutions being in alignment with Black community imperatives for advancing racial health equity [12,44]. As Israel and colleagues describe, “a community empowerment model transcends hierarchical, patriarchal, coercive, or violent conceptualizations of power and challenges the assumption that power is a zero-sum commodity” [15]. Community empowerment leverages a strengths-based lens, focused on community determination and the centering, harnessing, and resourcing of existing cultural strengths to create healing spaces [31]. Study findings offer strategies directly from Black community partners for increasing community agency, efficacy, and power in health research.

Study participants also expressed a desire to subvert research team roles common in dominant health research models. Community power building offers a unique opportunity for academic health researchers to assume alternative roles to support the advancement of racial health justice agendas and broader systems change. Such roles include (1) “the catalyst”, who serves as a change agent at the beginning of community change processes; (2) “the facilitator”, who assists bringing communities together; and (3) “the ally” and “the advocate”, who leverage their skill sets and capital to support community transformation [20]. Alternative roles are often indirect and more likely to ask communities “how to help, rather than making assumptions about what to do” [20] (p. 193). These alternative roles are centered around two-way communication and horizontal relationship building, in which both the academic health researcher and community partner work together to advance community change [20]. These roles are also intrinsically motivated, proactive, and not easily swayed by external contextual shifts like political pressures [20]. These roles also emphasize the importance of academic health researchers naming their own points of power and privilege [44]. By shifting their role to be in “power with” communities, academic health researchers can better be in coalition with Black community partners, build solidarity with community values, interests, and research priorities, and support the use of CER as an advocacy tool to advance structural change.

The small sample size (n = 12) could be considered a limitation of this study. This sample size was intentionally selected for two reasons. The first reason is that the study leveraged a purposive sampling strategy to obtain in-depth, information-rich cases about a specific social and cultural context. Given that the research objective was to gain in-depth information from research participants about their lived experiences as Black community partners in academic health research projects, a smaller sample size for interviews and focus groups was considered appropriate. Due to personal stories and lived experiences being unique to each storyteller, smaller sample sizes are common for narrative inquiry approaches [45]. The second reason for this sample size was data sufficiency. Data sufficiency seeks to address research questions efficiently, credibly, and ethically, while acknowledging the pragmatic constraints of reaching data saturation [46]. Given the limited scope and capacity constraints of the principal investigator’s doctoral dissertation research, choosing depth through more in-depth interviews with fewer study participants over breadth through a larger number of study participants was considered more appropriate.

## 5. Conclusions

This study offers a disciplinary critique of dominant forms of academic health research and alternative approaches for more equitable research engagement with a minoritized population. Insights from study findings can inform multi-level strategies for redistributing power in community-engaged health research processes. Authors offer the following recommendations for power shifting within community–academic partnerships involving Black communities below. These recommendations are guided by study results, the existing literature, and authors’ positionalities as critical health scholars and predominately Black scholar-activists.

At the intrapersonal level, individual academic health researchers must interrogate their own power, identity, and privilege, regardless of racial concordance with Black community partners and research participants. This can be achieved through continuous self-reflection of their positionality, which involves looking inward to reflect on how their unique social positions and worldviews create differing modes of advantage and disadvantage, as well as influence their motivations, approaches, and engagement with health research. Academic health researchers, particularly White academic health researchers, should also engage in cultural humility and consider taking alternative roles that are supportive of community desires and needs as opposed to leadership roles.

At the interpersonal level, academic health researchers must identify and implement strategies for redistributing power equitably across the entire research lifecycle between themselves, Black community partners, and Black research participants. This includes strategies that can shift agenda-setting, decision-making, and ideological power equitably to Black community partners. This can also include shifting ownership and management of knowledge, material, and cultural resources and power to Black community members. Additionally, it is critical that academic health researchers have a sustained, proactive commitment to advancing racial health equity. This includes a commitment to translating their research into social action to advance the community conditions in which Black community members are born, live, and age. This can look like leveraging data and research activities to inform community agenda-setting, policy changes, and the cultivation of healing spaces. Contemporary CER studies involving academic health researchers and Black community partners offer example strategies for attending to relational power asymmetries across research processes [47,48].

At the institutional level, academic health research institutions must recognize the critical social accountability role they play in their surrounding communities to promote population health. As anchors and hubs of influence in their communities, academic health research institutions must acknowledge that they are beholden to their communities—both through their organizational missions and visions, as well as through legal means. For example, U.S. academic health centers that have a tax-exempt status or receive federal funding are required by law to engage in certain community benefit activities, including community health needs assessments and provision of charitable care. Finally, academic health research institutions must engage in reconciliation efforts with their surrounding Black communities to redress historical and contemporary harms. Efforts must be context-specific and directly informed by community desires and needs. It is critical that the burden of antiracist change is not only held by individual academic health researchers. Academic health research institutions must demonstrate a sustained, proactive investment in racial justice and stay true to their antiracist commitments and declarations of racism as a public health crisis made in 2020 [49].

In alignment with the fourth generation of health disparities research and contemporary efforts to reimagine public health, critical actions must be taken at the systems level to both strengthen Black community capacity to engage in health research while simultaneously identifying and removing systemic barriers to health research engagement [50]. Systems change recommendations include but are not limited to the following:

- Building the research capacity of Predominately Black Institutions (PBIs) and Historically Black Colleges and Universities (HBCUs). This should be achieved through federal investment and implementing policy changes to ensure that more PBIs and HBCUs receive R1 “Very high research activity” designations.
- Investing in diversifying health workforce pathways, from K-12 education to academic health leadership, to increase the number of Black health researchers. This also includes closing the funding gap to ensure more Black principal investigators are funded to lead research on racial health equity.
- Identifying and removing systemic barriers in awareness, enrollment, and retention of Black research participants in health and biomedical research. This also includes improving public awareness of existing patient and community-engaged research advisory opportunities, such as the Food and Drug Administration’s Patient Representative Program and Patient-Centered Outcomes Research Institute’s Advisory Panels.
- Building the research capacity of Black communities to engage in community-led research (community efficacy). Building community efficacy requires identifying and removing procedural and bureaucratic injustices that many Black-led and managed community-based organizations and coalitions experience in accessing health research funding. It also requires innovative research funding models (like public–private partnerships, cross-agency federal funding approaches, federal challenges and prize competitions, and philanthropic social impact investments) to grow community-based health research and data infrastructure.

Identifying and addressing how racism as a system of power is operating in contemporary relations between Black communities and academic health research institutions is essential to advancing racial health equity. It is imperative that academic institutions and individual academic researchers move away from performative, reactive engagement with Black communities and move towards sustained commitments to achieving racial health equity. All academic researchers and research institutions must acknowledge that data and knowledge are a source of power. The data and knowledge produced from health research can be used as a tool to inform community action, equip Black communities with the knowledge, skills, and resources to protect their health and wellbeing, and create and implement racial health equity interventions. Centering diverse Black communities throughout health research processes and outcomes will enhance their abilities to create and sustain collective healing spaces.

## Figures and Tables

**Table 1:** Participant characteristics.

Participant Characteristic	n = 12	%
Race		
American Indian or Alaska Native	0	0.0
Asian	0	0.0
Black or African American	10	83.3
Middle Eastern or North African	0	0.0
Native Hawaiian or Other Pacific Islander	0	0.0
White	0	0.0
Multiracial or Multiethnic	2	16.6
Gender		
Cisgender Female	9	75.0
Cisgender Male	2	16.7
Nonbinary	1	8.3
Geographic Region		
Mid-Atlantic	2	16.7
Midwest	2	16.7
Northeast	1	8.3
Southeast	4	33.3
Southwest	1	8.3
West	2	16.7

**Table 2:** Visioning exercise (from interview guide).

Sub-Topic	Main Question
**Prompt:** Imagine you had unlimited power, resources, and money.	
Community Agency	What health topics would you want to research?
	If you were able to conduct research on the health topics you just described, what role would you want to play in that research?
	What types of data would you want to collect and analyze?
	What types of research products would you want to produce?
Community Efficacy	What types of resources would make it possible for you to conduct that research?
	What knowledge and skills would you need to conduct that research?
Community Solidarity	What role, if any, would you want academic researchers to play in that research?
	What support, if any, would you want academic researchers and research institutions to provide?

## Data Availability

Study documentation supporting the conclusions of this article will be made available by the authors on request.

## Supplementary Materials

The following supporting information can be downloaded at: https://www.mdpi.com/article/10.3390/ijerph22060921/s1, Table S1: Interview Guide; Table S2: Member Check Focus Group Guide.

## Abbreviations

The following abbreviations are used in this manuscript:

CER	Community-Engaged Research
CAP	Community–Academic Partnership

## References

[1] Wallerstein N., Duran B., Oetzel J.G., Minkler M. (2017). Community-Based Participatory Research for Health: Advancing Social and Health Equity.

[2] Key K.D., Furr-Holden D., Lewis E.Y., Cunningham R., Zimmerman M.A., Johnson-Lawrence V., Selig S. (2019). The Continuum of Community Engagement in Research: A Roadmap for Understanding and Assessing Progress. Prog. Community Health Partnersh. Res. Educ. Action.

[3] U.S. Department of Health and Human Services (2011). Principles of Community Engagement, Second Edition. https://ictr.johnshopkins.edu/wp-content/uploads/2015/10/CTSAPrinciplesofCommunityEngagement.pdf.

[4] Washington H.A. (2006). Medical Apartheid: The Dark History of Medical Experimentation on Black Americans from Colonial Times to the Present.

[5] Hoberman J. (2012). Black and Blue: The Origins and Consequences of Medical Racism.

[6] Roberts D. (2014). Killing the Black Body: Race, Reproduction, and the Meaning of Liberty.

[7] Washington H.A. (2021). Carte Blanche: The Erosion of Medical Consent.

[8] Williamson H.J., Chief C., Jiménez D., Begay A., Milner T.F., Sullivan S., Torres E., Remiker M., Samarron Longorio A.E., Sabo S. (2020). Voices of Community Partners: Perspectives Gained from Conversations of Community-Based Participatory Research Experiences. Int. J. Environ. Res. Public Health.

[9] Arnstein S. (1969). A Ladder of Citizen Participation. J. Am. Inst. Plan..

[10] Travers R., Pyne J., Bauer G., Munro L., Giambrone B., Hammond R., Scanlon K. (2013). ‘Community control’ in CBPR: Challenges experienced and questions raised from the Trans PULSE project. Action Res..

[11] Tchida C.V., Stout M. (2023). Disempowerment versus empowerment: Analyzing power dynamics in professional community development. Community Dev..

[12] Lukes S. (2021). Power: A Radical View.

[13] Gaventa J. (2006). Finding the Spaces for Change: A Power Analysis. IDS Bull..

[14] Gaventa J., Cornwall A. (2006). Challenging the Boundaries of the Possible: Participation, Knowledge and Power. IDS Bull..

[15] Israel B.A., Checkoway B., Schulz A., Zimmerman M. (1994). Health Education and Community Empowerment: Conceptualizing and Measuring Perceptions of Individual, Organizational, and Community Control. Health Educ. Q..

[16] Follett M.P. (1924). Creative Experience.

[17] Dowding K.M. (1996). Power.

[18] Purdy J.M. (2012). A Framework for Assessing Power in Collaborative Governance Processes. Public Adm. Rev..

[19] Mathie A., Cameron J., Gibson K. (2017). Asset-based and citizen-led development: Using a diffracted power lens to analyze the possibilities and challenges. Prog. Dev. Stud..

[20] Toomey A.H. (2011). Empowerment and disempowerment in community development practice: Eight roles practitioners play. Community Dev. J..

[21] Pierson-Brown T. (2022). It’s Not Irony, it’s Interest Convergence: A CRT Perspective on Racism as Public Health Crisis Statements. J. Law Med. Ethics.

[22] Lett E., Adekunle D., McMurray P., Asabor E.N., Irie W., Simon M.A., Hardeman R., McLemore M.R. (2022). Health Equity Tourism: Ravaging the Justice Landscape. J. Med. Syst..

[23] Delgado R., Stefancic J. (2023). Critical Race Theory. Vol. Fourth Edition: An Introduction.

[24] Bell D.A. (1980). Brown v. Board of Education and the Interest-Convergence Dilemma. Harv. Law Rev..

[25] Souto-Manning M. (2014). Critical narrative analysis: The interplay of critical discourse and narrative analyses. Int. J. Qual. Stud. Educ..

[26] Oudshoorn A., Sangster Bouck M., McCann M., Zendo S., Berman H., Banninga J., Le Ber M.J., Zendo Z. (2021). A critical narrative inquiry to understand the impacts of an overdose prevention site on the lives of site users. Harm Reduct. J..

[27] Solórzano D.G., Yosso T.J. (2002). Critical race methodology: Counter-storytelling as an analytical framework for education research. Qual. Inq..

[28] Iwelunmor J., Newsome V., Airhihenbuwa C.O. (2014). Framing the impact of culture on health: A systematic review of the PEN-3 cultural model and its application in public health research and interventions. Ethn. Health.

[29] Airhihenbuwa C.O. (1999). Of Culture and Multiverse: Renouncing “the Universal Truth” in Health. J. Health Educ..

[30] Airhihenbuwa C.O. (1989). Perspectives on AIDS in Africa: Strategies for prevention and control. AIDS Educ. Prev..

[31] Airhihenbuwa C.O. (1995). Health and Culture: Beyond the Western Paradigm.

[32] Airhihenbuwa C.O. (2007). Healing Our Differences: The Crisis of Global Health and the Politics of Identity.

[33] Birt L., Scott S., Cavers D., Campbell C., Walter F. (2016). Member checking: A tool to enhance trustworthiness or merely a nod to validation?. Qual. Health Res..

[34] Braun V., Clarke V. (2019). Reflecting on reflexive thematic analysis. Qualitative Research in Sport. Exerc. Health.

[35] Braun V., Clarke V., Hayfield N., Davey L., Jenkinson E., Bager-Charleson S., McBeath A. (2022). Doing Reflexive Thematic Analysis. Supporting Research in Counselling and Psychotherapy: Qualitative, Quantitative, and Mixed Methods Research.

[36] Roberts R.E. (2020). Qualitative Interview Questions: Guidance for Novice Researchers. Qual. Rep..

[37] Laverack G. (2006). Improving Health Outcomes through Community Empowerment: A Review of the Literature. J. Health Popul. Nutr..

[38] Foucault M. (2019). Power: The Essential Works of Michel Foucault 1954–1984.

[39] Foundation R.W.J. (2023). Building Community Power to Advance Health Equity. https://www.rwjf.org/en/our-vision/focus-areas/Features/building-community-power-to-advance-health-equity.html.

[40] Popay J., Whitehead M., Ponsford R., Egan M., Mead R. (2020). Power, control, communities and health inequalities I: Theories, concepts and analytical frameworks. Health Promot. Int..

[41] Iton A., Ross R.K., Tamber P.S. (2022). Building Community Power to Dismantle Policy-Based Structural Inequity in Population Health: Article describes how to build community power to dismantle policy-based structural inequity. Health Aff..

[42] Speer P.W., Gupta J., Haapanen K., Balmer B., Wiley K.T., Bachelder A. (2020). Developing Community Power for Health Equity: A landscape Analysis of Current Research and Theory.

[43] Haugaard M. (2012). Rethinking the four dimensions of power: Domination and empowerment. J. Political Power.

[44] Heller J.C., Fleming P.J., Petteway R.J., Givens M., Pollack Porter K.M. (2023). Power up: A call for public health to recognize, analyze, and shift the balance in power relations to advance health and racial equity. Am. J. Public Health.

[45] Subedi K.R. (2021). Determining the Sample in Qualitative Research. Online Submiss..

[46] Suri H. (2011). Purposeful sampling in qualitative research synthesis. Qual. Res. J..

[47] Antoine-LaVigne D., Hayes T., Fortenberry M., Ohikhuai E., Addison C., Mozee S., McGill D., Shanks M.L., Roby C., Jenkins B.W.C. (2023). Trust and biomedical research engagement of minority and under-represented communities in Mississippi, USA. Int. J. Environ. Res. Public Health.

[48] Denyse T., Martin K.J., Stanton A.L. (2022). The Ubuntu Approach in Project SOAR (Speaking Our African American Realities): Building a robust community-academic partnership and culturally curated focus groups. Soc. Sci. Med..

[49] American Public Health Association (2025). Racism Declarations.

[50] Thomas S.B., Quinn S.C., Butler J., Fryer C.S., Garza M.A. (2011). Toward a Fourth Generation of Disparities Research to Achieve Health Equity. Annu. Rev. Public Health.

