# Supplementary material for: Reimagining Partnerships Between Black Communities and Academic Health Research Institutions: Towards Equitable Power in Engagement
Source: Int J Environ Res Public Health. 2025 Jun 10;22(6):921. doi: 10.3390/ijerph22060921 (PMC12193660; doi:10.3390/ijerph22060921)
Supplement: Supplementary file 1 [file ijerph-22-00921-s001.zip › ijerph-3648649-supplementary.pdf]

<u>Table S1. Interview Guide</u>

<table>
<tr><td colspan="2">Topic: Self and Community Identity
Purpose: To better understand who community partners are and the communities they identify with.</td></tr>
<tr><td>Sub-Topic</td><td>Lead Question and Probes</td></tr>
<tr><td>Self-Identity</td><td>What personal identities are important to you and your story?</td></tr>
<tr><td>Community Identity</td><td>What communities do you have a sense of belonging to?</td></tr>
<tr><td>Black Community Meaning Making</td><td>When you hear the phrase ‘Black community’, what comes to mind?

What does ‘Black community(ies)’ mean to you?</td></tr>
<tr><td colspan="2">Topic: Interest in Health Research
Purpose: To better understand community partners’ motivations for involvement in health research.</td></tr>
<tr><td>Sub-Topic</td><td>Lead Question and Probes</td></tr>
<tr><td>Motivation for Research Involvement</td><td>What motivated you to get involved in health research?</td></tr>
<tr><td>Motivation for Advancing Racial Health Equity</td><td>Why is improving the health of Black people important to you?</td></tr>
<tr><td>Perception of Black People’s Relationship to Research</td><td>What are common beliefs about Black people’s relationship to research? What are some misconceptions?</td></tr>
<tr><td colspan="2">Topic: Reimagining Health Research
Purpose: To gain insight into community partners’ perspectives on how their communities can have equitable power in shaping health research.
Prompt/Invitation: Imagine you had unlimited power, resources, and money.</td></tr>
<tr><td>Sub-Topic</td><td>Lead Question(s)</td></tr>
<tr><td>Community Agency</td><td>What health topics would you want to research?

If you were able to conduct research on the health topics you just described, what role would you want to play in that research?

What types of data would you want to collect and analyze?

What types of research products would you want to produce?</td></tr>
<tr><td>Community Efficacy</td><td>What types of resources would make it possible for you to conduct that research?</td></tr>
</table>

	What *knowledge and skills* would you need to conduct that research?
**Community Solidarity**	What *role*, if any, would you want academic researchers to play in that research?<br><br>What *support*, if any, would you want academic researchers and research institutions to provide?

Table S2. Member Check Focus Group Guide

Section	Question
**Section I. Presentation of Reflexive Thematic Analysis (RTA) process of condensing data and quality assurance actions (internal validity measures and research reflexivity process)**	Do you have any questions or comments about the data analysis process before we proceed to the initial findings?
**Section II. Presentation of Initial Findings (based on *RTA Step 4: Review of Potential Themes*)**	***Pause after each potential theme is shared.***<br><br>Do these findings resonate with you?<br><br>Is there anything you would add, remove, or change?
**Section III. Input on Research Products**	Are there any alternative research products (beyond a manuscript) you would like to see come out of this research?<br><br>Are there any alternative in-person or digital outlets/forums (beyond an academic conference) that you would like these research findings shared?
